# Penetration of Milk-Derived Antimicrobial Peptides into Phospholipid Monolayers as Model Biomembranes

**DOI:** 10.1155/2013/914540

**Published:** 2013-12-17

**Authors:** Wanda Barzyk, Ewa Rogalska, Katarzyna Więcław-Czapla

**Affiliations:** ^1^Jerzy Haber Institute of Catalysis and Surface Chemistry, Polish Academy of Sciences, Niezapominajek Street 8, 30-239 Cracow, Poland; ^2^UMR 7565 CNRS SRSMC, Université de Lorraine, Faculté des Sciences et Technologies, BP 239, 54506 Vandoeuvre-lès-Nancy Cedex, France; ^3^Department of Physical Chemistry and Electrochemistry, Faculty of Chemistry, Jagiellonian University, Ingardena 3, 30-060 Cracow, Poland

## Abstract

Three antimicrobial peptides derived from bovine milk proteins were examined with regard to penetration into insoluble monolayers formed with 1,2-dipalmitoyl-*sn*-glycero-3-phosphocholine (DPPC) or 1,2-dipalmitoyl-*sn*-glycero-3-phospho-*rac*-(1-glycerol) sodium salt (DPPG). Effects on surface pressure (Π) and electric surface potential (Δ*V*) were measured, Π with a platinum Wilhelmy plate and Δ*V* with a vibrating plate. The penetration measurements were performed under stationary diffusion conditions and upon the compression of the monolayers. The two type measurements showed greatly different effects of the peptide-lipid interactions. Results of the stationary penetration show that the peptide interactions with DPPC monolayer are weak, repulsive, and nonspecific while the interactions with DPPG monolayer are significant, attractive, and specific. These results are in accord with the fact that antimicrobial peptides disrupt bacteria membranes (negative) while no significant effect on the host membranes (neutral) is observed. No such discrimination was revealed from the compression isotherms. The latter indicate that squeezing the penetrant out of the monolayer upon compression does not allow for establishing the penetration equilibrium, so the monolayer remains supersaturated with the penetrant and shows an under-equilibrium orientation within the entire compression range, practically.

## 1. Introduction

### 1.1. Structure of Antimicrobial Peptides and Their Action on Pathogenic Cell Membranes

Antimicrobial peptides (AMPs), named also as antibiotic or host defense peptides (HDPs), are evolutionarily conserved components of the innate immune response of a variety of organisms, such as amphibians, invertebrates, plants, and mammals [1]. To date, ca. 2000 different AMPs have been identified or predicted [2, 3]. Many AMPs exhibit a broad spectrum of antimicrobial activity against Gram-positive and Gram-negative bacteria, fungi, parasites, enveloped viruses and cancerous cells [4–7]. In contrast to conventional antibiotics, AMPs appear to be bacteriocidal (bacteria killer) instead of bacteriostatic (bacteria growth inhibitor). They can destroy bacteria within minutes with the rate being faster than the bacteria growth rate [8]. Therefore, AMPs are recognized as potent source of pharmaceuticals for the treatments of multidrug-resistant microorganisms [1–21]. To date, several AMPs are in clinical trials [1, 5, 22]. Interest in AMPs is being constantly increasing during the last ten years which resulted in a number of publications on structure, bioactivity and mechanisms of action of particular AMPs on microbial or model cell membranes. These investigations are reviewed in a number of articles [2, 4–21].

AMPs show an extraordinary structural diversity of primary and secondary structure, and the latter is often different in solutions and lipidic environment [20, 22, 23]. AMPs mostly range between 10 and 40 [3, 8] of amino acid residues (although there are shorter or larger peptides classified as AMPs). Most of the AMPs share two common fundamental features—they are cationic and amphiphatic [2, 11–13]. The cationic charge is contributed by positive amino acids (arginine, lysine, and also—in acidic environment—histidine) from 2 to 9 per peptide molecule [2, 3]. The crucial property of AMPs' structure is a large proportion of hydrophobic amino acid residues (≥30% [2]) which are spatially organized in discrete sectors of the molecule, making it amphiphatic. The key property of AMPs is selective toxicity to microbial or cancerous cell membranes with no significant toxicity to native (host) cells. The selectivity is driven by a different charge of outer leaflet of microbial (negative) and mammalian/plant (neutral) cell membranes [1, 5, 6, 8, 11]. The increase in hydrophobicity of AMP is strongly correlated with a loss of its selectivity [11].

There is no common molecular mechanism of action of AMPs—it depends on the nature of the peptide, the membrane lipid composition and the peptide/lipid ratio [1, 2, 8, 11]. The mechanism comprises several stages which are not yet fully understood, despite extensive studies. The necessary step is peptide's association with membrane lipids which results in long-range defects. The different molecular mechanisms postulated (such as barrel-stave or toroidal/wormhole pore formation, aggregate channel formation or surfactant-like interactions [1, 2, 8, 11–14, 16, 19]) assume that aggregation/oligomerization of AMP in the cytoplasmic membrane is the necessary step leading to the membrane lysis.

### 1.2. Milk-Derived AMPs

Antibacterial properties of milk have been known for a long time. Bovine milk proteins are a natural reservoir of bioactive peptides which are released during gastrointestinal digestion of milk or its fermented products. So far, several peptides released from milk proteins have been recognized as having a wide spectrum of antimicrobial activities [24–35]. These AMPs are regarded as nontoxic for mammalian cells; therefore, they are considered as potent drugs, food biopreservatives, and/or supplements in functional foods [35]. Interest in milk AMPs is constantly growing. Those derived from the major milk proteins (caseins, *α*-lactalbumin, and *β*-lactoglobulin) have been recently reviewed in [35]. The most often investigated milk-derived AMP is so far bovine lactoferricin (LfcinB), originally derived from lactoferrin (the minor milk protein) as 25 aminoacid peptide [36–39]. LfcinB shows an extraordinarily broad range of bioactivities, including antibacterial, antifungal, antiparasitic, and anticancerous [39]. Much less is known on other milk-derived AMPs. One of the most promising of them is lactophoricin-I which of structure (primary and secondary) is completely different from lactoferricin B (LfcinB). Lactophoricin-I is announced as the first AMP derived from lactophorin [32]—the minor milk protein. Although, there are several reports on lactophoricin-I, describing its production [32, 33, 40], structure [32–34, 40, 41], and interactions with model biomembranes, (such as supported lipid bilayers [33] and micelles [41], as well as antimicrobial activities* in vitro *[32, 41]), further investigations on this peptide and other milk-derived AMPs are required to recognize their potential applications.

### 1.3. Studies of AMP Interactions with Phospholipid Monolayers

Phospholipid monolayer spread at the air/aqueous solution interface is considered as a halfmembrane and recommended as the simplest model of cell membrane [42–45]. Lateral pressure (Π) of biomembranes is put forward in the range of 30–35 mN/m [46, 47], although, owing to different lipid compositions of various biomembranes, their pressure may fall in the lower range, Π > 20 mN/m. It is thought that the density range of biomembranes corresponds to mixture of liquid expanded (LE) and liquid condensed (LC) phospholipid phases or to a LC phase.

The monolayer model of biomembrane enables overcoming some serious limitations created by the more advanced models [44], such as phospholipid unilamellar vesicles (SUV or LUV) creating theoretical problems with their surface curvature, or supported phospholipid bilayers which of structures are strongly influenced by the support. The monolayer model is convenient for investigations on initial peptide-lipid interactions involving peptide's association to lipid head groups which is followed by a partial embedding in the hydrophobic region; these stages are found sufficient for antimicrobial action of numerous AMPs.

There is a number of papers in which kinetics of AMP association with phospholipid monolayers was investigated under stationary diffusion conditions to acquire the so-called penetration profiles of the surface pressure, ΔΠ versus Π_init_ [48–53]. Increase of the surface pressure, ΔΠ, upon the penetration process is attributed to compression of the lipid component owing to the amphiphile's insertion [46]. The penetration profiles, ΔΠ versus Π_init_, of numerous AMPs are found linear [48–51], likewise those measured for insertion of bioactive peptides and proteins [47, 54, 55]. Linearity of the ΔΠ versus Π_init_ profiles is attributed to linear increase of the surface pressure with mole fraction of the inserted amphiphile—the dependency is valid only for low amphiphile concentrations [46]. On the other hand, also nonlinear ΔΠ versus Π_init_ profiles have been reported for some AMPs [52, 53] or bioactive peptides [47, 56–58]. Interpretation of the profiles is complicated by the fact that the surface pressure may change with degree of peptide insertion [46] or with 2D phase composition of the monolayer [47]. Investigations on stationary penetration of AMPs are most often combined with compression isotherms of pure peptide and peptide-lipid mixed monolayers [59, 60].

In the present work, three antimicrobial bovine milk-derived peptides called E-5-K, L-16-Y, and N-23-T (the names formed of symbols of the first and the last aminoacid residue and their total number) were tested with respect to penetration to insoluble monolayers formed with 1,2-dipalmitoyl-*sn*-glycero-3-phosphocholine (DPPC) or 1,2-dipalmitoyl-*sn*-glycero-3-phospho-*rac*-(1-glycerol) sodium salt (DPPG). The two type monolayers of well-documented properties (DPPC [61–64], DPPG [62, 65–71]) serve us as crude model of mammalian (neutral) and bacteria (negative) cell membranes, respectively. The most promising of the investigated peptides is N-23-T, recently named lactophoricin-I [32, 40, 41]. It shows an amphiphatic structure [40, 41] and a range of antimicrobial activities [32]. The two other milk AMPs were chosen with respect to their different charge as compared to N-23-T (cf.  Table 1).

The main purpose of this investigation is to characterize the molecular interactions in terms of the surface pressure, Π, and the electric surface potential, Δ*V*. The Π measurement supplies the direct information on free energy of interactions between lipid monolayer and the penetrant. The surface potential measurement, scarcely used, so far, in investigation of AMPs' penetration into lipid monolayers [72–74], supplies information on state of ordering the monolayer. The effects on Π and Δ*V* upon the peptides' penetration were examined under stationary diffusion conditions in relation to: (1) the initial density of phospholipid monolayer characterized by the equilibrium surface pressure, Π_init_, (2) type of phospholipid (DPPC or DPPG) and (3) the peptide's concentration. The ΔΠ and Δ*V* effects upon the penetrant's association with the film are discussed in terms of mutual interactions and disordering the monolayer. Dependence of the penetration effects on the film density is discussed in terms of fit ability of the penetrant's amphiphatic structure to density of phospholipid monolayer. For lactophoricin-I, compression isotherms (Π-*A* and Δ*V*-*A*) on subphase containing this peptide are also discussed. The investigations are focused on verifying whether the Π and Δ*V* effects measured upon compression of the lipid monolayer on subphase containing the penetrant differ from those obtained under stationary diffusion conditions.

## 2. Materials and Methods

### 2.1. Materials

1,2-dipalmitoyl-*sn*-glycero-3-phosphocholine (DPPC, ≥99%) and 1,2-dipalmitoyl-*sn*-glycero-3-phospho-*rac*-(1-glycerol) sodium salt (DPPG, 99%) were from Sigma. The peptides were originally isolated from the sequence of bovine milk proteins: 5 residues peptide from *α*-lactalbumin was named E-5-K, 16 residues peptide from *α*-s2 casein was named L-16-Y, and 23 residues peptide from component-3 of proteose peptone (PP3) was named N-23-T or lactophoricin-I [32] (the latter is alternatively denoted in the literature as LPcin-I [40, 41]). More information on synthesis and purification of these peptides is given in [32, 33, 75] and on the structure and antimicrobial activities *in vitro* in [26, 31–34]. The available data on physicochemical properties of the peptides are comprised in Table 1. The net charge predicted using the “Peptide Property Calculator” software of CS Bio Co. for E-5-K is 0 in the pH range of 5.5–8, for L-16-Y is +4 in the pH range of 4–8, and for N-23-T is +2.1 in the pH range of 7-8, while under the used pH of 5.6 it adopts the value of +3.

The used Milli-Q water was of the electric conductivity (in contact with CO_2_) of ca. 0.8 *μ*S/cm and of the surface tension of 72.8 mN m^−1^. The phospholipid stock solutions were prepared with spectrophotometric grade chloroform (Aldrich, A.C.S.).

### 2.2. The Penetration Measurement under Stationary Diffusion Conditions

These measurements were performed in a Petri dish of inner diameter of 2*r* = 9.5 cm. Since the peptides were available in small amounts, the subphase volume was minimized to 20 mL. The measurement procedure was as follows. Phospholipid monolayer was spread at the air/water interface from ca. 1 mg/mL DPPC or DPPG solution in 10% ethanol in CCl_4_ (using a Hamilton syringe) up to reaching the desired surface pressure, Π_init_, established after ca. 10 min. equilibration of the film. Next, aqueous peptide solution (100–1000 *μ*L) was injected beneath the phospholipid monolayer to adjust the peptide's desired concentration in the range of 5 × 10^−7^–1 × 10^−5^ mol dm^−3^. The crucial step of these experiments was averaging peptide's concentration just after its injection, performed on the way securing against a disturbance of the phospholipid film. It was achieved by means of a glass stirrer (mounted at the bottom of the cell) which was put rotated during ca. 5 s after the injection. Next, the penetration process was investigated by measuring time evolution of surface pressure, Π-*t*, and electric surface potential, Δ*V*-*t*, during 60 min.

The surface pressure (Π) and the electric surface potential (Δ*V*) measurements were performed using the KSV 5000 Langmuir balance and the KSV 1000 SPD surface potential meter (KSV Ltd., Helsinki). The surface pressure (Π) was measured with a platinum Wilhelmy plate (perimeter 3.94 cm) and the electric surface potential (Δ*V*) with a gold plated vibrating plate (VP)—not perforated. The counter electrode for the Δ*V* measurement was a silver, silver chloride half cell (Ag, AgCl/3 M KCl) manufactured by the Radiometer firm. The measuring sensors and the cell were placed together in a Plexiglas box thermostated at 20°C.

### 2.3. The Compression Isotherms, Π-*A* and Δ*V*-*A*

A Teflon trough (15 cm × 58 cm × 1 cm) with two hydrophilic Delrin barriers (symmetric compression) was used in compression isotherm experiments. The system was equipped with an electrobalance and a platinum Wilhelmy plate (perimeter 3.94 cm) as the surface pressure sensor. Surface potential was measured using the KSV Spot 1 with the gold plated vibrating plate. The counter electrode for the measurement—supplied by the producer—was made of a stainless steel. The apparatus was closed in a Plexiglas box thermostated at 20°C. All solvents used for cleaning the trough and the barriers were of analytical grade. Calibrated solutions of DPPC and DPPG in chloroform (concentration around 0.5 mg mL^−1^) were used for spreading (with a microsyringe of Hamilton Co., USA) lipid monolayers on the peptide's solution subphase. After the equilibration time of 20 min, the films were compressed at the rate of 2.5 mm min^−1^ barrier^−1^ by two symmetrically moving barriers. A PC computer and KSV software were used to control the experiments. Each compression isotherm was performed at least three times. The standard error was ±0.5 Å^2^ with mean molecular area, ±0.2 mN m^−1^ with surface pressure, and ±5 mV with surface potential measurements.

## 3. Results and Discussion

### 3.1. Surface Activity of the Milk-Derived Peptides at the Air/Water Interface

Activity of the peptides at the air/water interface was investigated by injecting an amount of the original peptide's solution into water sample to reach the desired concentration after stirring. It was found that the only one of the peptides, lactophoricin-I (N-23-T), increases significantly the surface pressure at the air/solution interface; that is, solutions of 1 × 10^−6^, 5 × 10^−6^ and 1 × 10^−5^ mol N-23-T dm^−3^ showed the equilibrium surface pressure of 12.2, 16.7, and 19.5 mN m^−1^, respectively, and about 90% of the Π values were established during 30 min after the peptide injection. Lactophoricin-I produced also a high change of the electric surface potential, Δ*V*, which increased between 400 and 1100 mV, depending on the concentration. In contrast, L-16-Y and E-5-K peptides did not show surface activity at the air/solution interface (i.e., Π in the range between −2.5 and +0.5 mN m^−1^ and Δ*V* in the range of 100–600 mV were measured at their concentrations in the range of 1 × 10^−6^–1 × 10^−5^ mol dm^−3^). Noteworthy, the surface potential of the nearly neutral peptide E-5-K started at a slightly negative value in the range of −200 to −50 mV (depending on the concentration) and increased by ca. 300 mV during 60 min. The surface activity of N-23-T at the air/solution interface (in contrast to L-16-Y and E-5-K) should be ascribed to the relatively greatest amount of hydrophobic residues in the peptide chain and in particular to situating all of them in outer sphere of the molecule (cf.  Table 1).

### 3.2. Compression Isotherms of DPPC and DPPG Monolayers on Lactophoricin Containing Subphase

**Figure 1:**
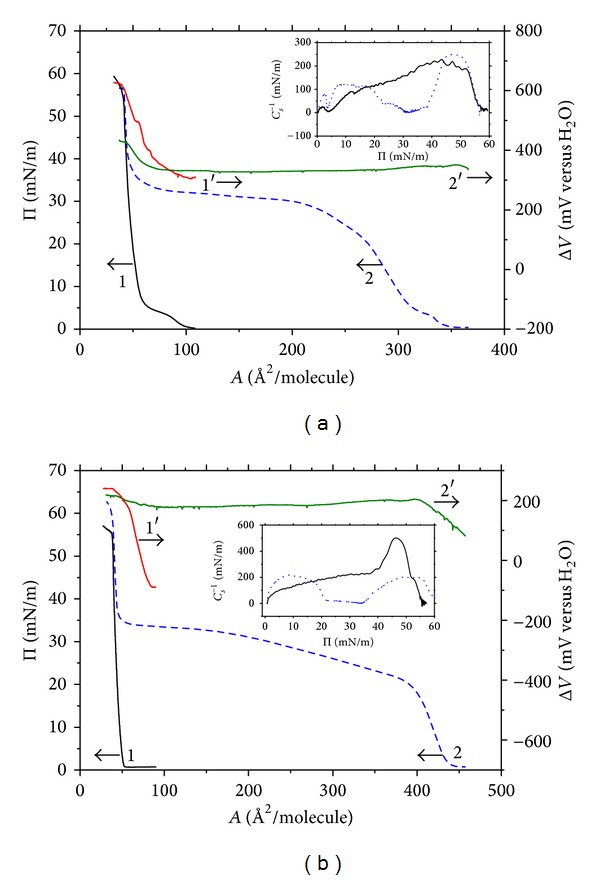
Compression isotherms of surface pressure, Π versus *A*, (curves 1 and 2) and electric surface potential, Δ*V* versus *A* (curves 1′ and 2′) of: (a) DPPC and (b) DPPG monolayers spread on subphase of water (curves 1 and 1′) and lactophoricin (N-23-T) solution, 1 *μ*M (curves 2 and 2′); “*A*” denotes area per the phospholipid molecule. The inserts show the surface compression modulus (*C*_*s*_^−1^) calculated according to (1) as a function of Π of the pure lipid monolayer (solid lines) and that spread on lactophoricin solution (dotted lines).

The surface pressure versus area (Π-*A*) and electric surface potential versus area (Δ*V*-*A*) isotherms registered upon compression of DPPC and DPPG monolayers on pure water and the subphase containing 1 × 10^−6^ mol dm^−3^ lactophoricin-I are shown in Figures 1(a) and 1(b).

**Figure 2:**
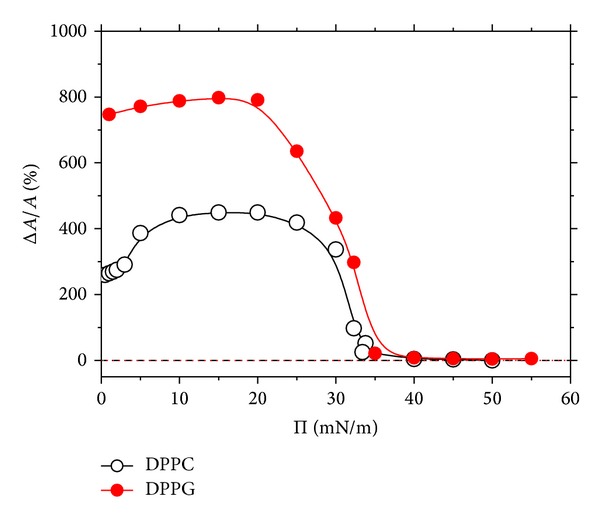
Effects of expanding DPPC and DPPG monolayers by lactophoricin-I (N-23-T). The Δ*A*/*A* (%) denotes the relative increase of area per the phospholipid molecule due to the peptide's penetration. It is evaluated (with the accuracy below 2%) by comparing the Π-*A* isotherms presented in Figures 1(a) and 1(b) at the same Π and plotted as a function of the Π. The peptide's concentration is 1 *μ*M.

One may notice in Figures 1(a) and 1(b) the huge expanding effect exerted by lactophoricin-I both on DPPC and DPPG monolayers. The percentage increase of area per the phospholipid molecule, Δ*A*/*A*, upon the peptide insertion is presented in Figure 2 as a function of Π. These Δ*A*/*A* versus Π dependencies were evaluated from the isotherms registered on the peptide containing subphase and pure water by comparing areas at identical Π values. Note that the expansion exerted by lactophoricin-I on DPPG monolayer (by ca. 800%) is about twice greater than that on DPPC, indicating that electrostatic interactions of the positively charged peptide with negatively charged phosphoglycerol groups drag more peptide into the monolayer as compared to neutral DPPC. On the other hand, a great expansion of DPPC monolayer by the peptide (by ca. 400%) indicates competitive adsorption of N-23-T at the air/water interface (cf.  Section 3.1). The adsorbed peptide, owing to its notable charge, may form highly spread 2D domains (Table 1). Taking the above in mind, the 10 < Π < 20 mN m^−1^ range corresponding to the maximum expanding effect is to be ascribed to the coexistence of 2D peptide domains with 2D phospholipid domains, LC. The latter may be to an extent perturbed by the peptide-lipid interactions, in particular in case of DPPG monolayer for which, also, formation of a mixed lipid-peptide 2D phase cannot be excluded. In the higher Π range, 20 < Π < 35 mN m^−1^, the expanding effect is steeply falling down to near zero which indicates collapse of the peptide's domains in the Π range. Noteworthy, the Π-*A* isotherms obtained by us for the DPPC- and DPPG-lactophoricin-I systems show a great analogy to those presented by Neville and coworkers [76, 77] for the penetration of cathelicidin peptide, LL37, into DPPC and DPPG monolayers. Interestingly, the isobaric insertion experiments (Δ*A*/*A* versus time measurements) [77] revealed a notable expanding effect of LL37 on DPPG (up to ca. 180%) which was not revealed, at all, for the DPPC/LL37 system, although the Π-*A* isotherm indicates it. The discrimination effect of LL37 in favour of the negative monolayer is supported by the results of the concurrent measurements using epifluorescence microscopy [77] and surface synchrotron X-ray scattering [76, 77] techniques which showed a great (progressing with time) structural change of DPPG monolayer upon LL37 penetration, opposite to DPPC monolayer; the latter did not show any significant structural change in contact with LL37. The authors [76] postulate the formation of a mixed DPPG-LL37 2D phase which undergoes an extra 2D phase transition indicated by a flat hump in the Π-*A* isotherm. There are, however, no other pieces of evidence for the formation of a mixed phase in the DPPG/LL37 or DPPG/lactophoricin systems, so far.

**Figure 3:**
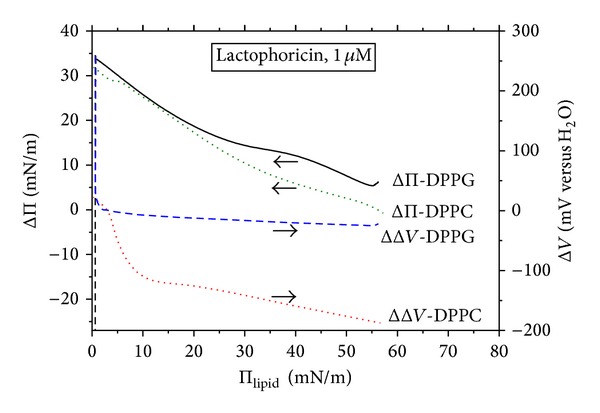
Effects of penetration of lactophoricin-I (N-23-T) into DPPC and DPPG monolayers on surface pressure (Π) and electric surface potential (Δ*V*); the ΔΠ and the ΔΔ*V* values calculated as difference in the Π and the Δ*V*ordinate of the isotherms registered on lactophoricin-I (N-23-T) solution (1 *μ*M) and the pure water subphase (cf. Figures 1(a) and 1(b))—at identical cross-sectional areas (*A*) per the phospholipid molecule, are plotted as a function of surface pressure of the pure phospholipid monolayer, Π_lipid_.

Figure 3 shows the penetration effects calculated by comparing the Π and Δ*V*ordinates of the isotherms registered in the presence and absence of the penetrant, respectively. The ΔΠ and ΔΔ*V* differences at the same *A* areas are displayed as a function of the surface pressure of the pure phospholipid monolayer, Π_lipid_. The ΔΠ versus Π_lipid_ dependencies shown in Figure 3 are counterparts of the so-called penetration profiles measured under stationary diffusion conditions as the surface pressure increases, ΔΠ, as a function of the initial Π value. However, it should be stressed here that the profiles derived from the compression isotherms correspond to squeezing penetrant out of the monolayer, opposite to the profiles measured directly upon the penetration process under stationary diffusion conditions. One may notice in Figure 3 that the maximum ΔΠ effects determined for DPPC/N-23-T and DPPG/N-23-T systems are comparable, amounting to 32 and 34 mN m^−1^, respectively. The ΔΠ effect decreases with increasing the lipid density, reaching the minimum (of ca. −1 mN m^−1^ for the DPPC/N-23-T system and ca. 6 mN m^−1^ for the DPPG/N-23-T) close to the collapse point. The ΔΠ versus Π_lipid_ dependencies presented in Figure 3 show a slight departure of linearity, a greater one for the DPPG/N-23-T than for DPPC/N-23-T system. The positive in sign ΔΠ effects within almost entire compression range indicates the excess attractive interactions of the both lipids with the penetrant. The corresponding ΔΔ*V* versus Π_lipid_ dependencies show much greater differentiation with respect to the phospholipid type as compared to the ΔΠ versus Π_lipid_ (Figure 3). The ΔΔ*V* versus Π_init_ dependencies decline within the entire compression range and in majority of it adopt values negative in sign. The ΔΔ*V* minimum reached close to the collapse point is ca. −200 mV for the DPPC/N-23-T system and ca. −20 mV for DPPG/N-23-T. The positive ΔΔ*V* effects are measured only within the lowest density range of Π < 3 mN m^−1^ in which DPPC and DPPG monolayers are highly expanded by the peptide (i.e., ca. 3 times in case of DPPC and ca. 8 times in case of DPPG). Such expansion allows for orientational freedom of the lipids. Accordingly, the higher expansion of DPPG monolayer, as compared to DPPC, is accompanied by the much less negative ΔΔ*V* effect. Since the net negative ΔΔ*V* effect is caused by the positively charged peptide, it is attributed to disorienting acyl chains of the phospholipid by the penetrant. This explanation is in accord with the commonly accepted view [78–80] that decrease in inclination of hydrocarbon chains with respect to the interface diminishes their contribution to the surface potential [80].

Inserts in Figures 1(a) and 1(b) show courses of the compressibility modulus,  *C*_*s*_^−1^, defined as [81, 82]

(1) $$\begin{array}{c} \;C_{s}^{-1}=-A{\left(\frac{\partial \Pi }{\partial A}\right)}_{T}, \end{array}$$

calculated from the Π-*A* isotherms. According to Davies and Rideal [81], value of *C*_*s*_^−1^ is indicative of 2D phase composition; namely, the range of 12.5 < *C*_*s*_^−1^ < 50 mN m^−1^ corresponds to liquid expanded phase (LE, chain disordered) and of 100 < *C*_*s*_^−1^ < 250 mN m^−1^, to liquid condensed phase (LC, chain ordered); (some authors [58] distinguish also the intermediate range of 50 < *C*_*s*_^−1^ < 100 mN m^−1^ ascribed to the L1 phase). Increase in *C*_*s*_^−1^ corresponds to increase of rigidity (i.e., decrease of compressibility) of the monolayer [82]. The *C*_*s*_^−1^ versus Π courses of DPPC monolayers (insert of Figure 1(a)) shows consistently local maximum at Π ca. 2.5 mN m^−1^ which is ascribed by us to the maximum density of LE-DPPC phase. The following minimum *C*_*s*_^−1^ versus Π within the 3 < Π < 7 mN m^−1^ range is ascribed to coexistence of LE and LC phases of DPPC. In the range corresponding to the greatest expansion exerted by lactophoricin-I on the phospholipid monolayers, 8 < Π < 20 mN m^−1^ (cf. Figure 2), the *C*_*s*_^−1^ versus Π dependency of the penetrated DPPC monolayer shows a slightly greater rigidity as compared to pure DPPC monolayer. It indicates the peptide's interactions with LC phase of DPPC. Just the opposite occurs within the 20 < Π < 35 mN m^−1^ range corresponding to the fall down of the expanding effect (cf. Figure 2); that is, the *C*_*s*_^−1^ versus Π successively falls down to the minimum of ca. 2 mN m^−1^ at Π ca. 31 mN m^−1^ that the minimum reflects the inflection point in the Π-*A* isotherm of the penetrated DPPC monolayer within the wide plateau region. The latter indicates a 2D phase transition (or possibly two consecutive ones) which may be ascribed to the peptide itself and, possibly, to a mixed peptide-lipid 2D phase. This hypothesis is based on the fact that monolayers of many AMPs on water subphase show a 2D transition in the 20 < Π < 30 mN m^−1^ range and collapse at Π ca. 30 mN m^−1^ [44, 49]. Collapse of DPPC monolayer on water subphase and 1 × 10^−6^ mol dm^−3^ lactophoricin-I solution occurs at Π values of 55.2 and 55.1 mN m^−1^, respectively, which correspond to the areas of 40 and 42 Å^2^ per lipid molecule [75]. These parameters indicate that lactophoricin-I has been squeezed from the monolayer before its collapse. A slight difference in the corresponding *C*_*s*_^−1^ versus Π dependencies within the 45 < Π < 52 mN m^−1^ range implies very slight interactions of phophocholine groups with the squeezed peptide.

Courses of the *C*_*s*_^−1^ versus Π dependencies calculated for DPPG monolayers (insert of Figure 1(b)) not only significantly differ from that for DPPC but also show a wide minimum within the 20 < Π < 35 mN m^−1^ range which is ascribed to squeezing the penetrant out of the monolayer (cf. Figure 2). The *C*_*s*_^−1^ values close to zero in this range indicate enormously great compressibility (fluidity) of the monolayer as the result of squeezing the peptide's domains. Since pure DPPG monolayer does not show LE/LC phases coexistence at pH ca. 5.6, at 20°C [65, 66], its *C*_*s*_^−1^ increases successively from zero to the maximum of ca. 500 mN m^−1^ ascribed to the 2D solid (S). Interestingly, in the range of the highest expansion of DPPG monolayer by lactophoricin-I, 8 < Π < 20 mN m^−1^ (cf. Figure 2), the penetrated monolayer shows local *C*_*s*_^−1^ versus Π maximum of ca. 210 mN m^−1^. It may be ascribed to LC-DPPG phase perturbed by the peptide. Just the opposite occurs in the range of falling down the expansion effect, 20 < Π < 35 mN m^−1^, wherein the *C*_*s*_^−1^ versus Π dependency shows the wide minimum of significantly different shape as compared to that of DPPC (Figure 1(b)). The collapse point of DPPG monolayers on water subphase and lactophoricin-I solution falls at the notably different Π values, of 53.1 and 58.1 mN m^−1^, corresponding to 40 Å^2^ and 41 Å^2^ per molecule, respectively [75]. These parameters indicate notable interactions of phosphoglyceride groups with lactophoricin-I squeezed into the subsurface region. These interactions are revealed also in shape of the *C*_*s*_^−1^-Π dependencies within the 40 < Π < 50 mN m^−1^ range, by notable lowering of *C*_*s*_^−1^ value of the penetrated DPPG monolayer relative to pure DPPG, that is, the decrease of *C*_*s*_^−1^ from ca. 500 to ca. 200 mN m^−1^ (c.f., Figure 1(b)) reflecting a significant increase of the monolayer's fluidity (i.e., softening the monolayer owing to interactions with the squeezed peptide). Since, the *C*_*s*_^−1^ value of 200 mN m^−1^ is typical of a liquid condensed 2D phase [81], we conclude that presence of the squeezed peptide does not allow for formation of 2D solid phase of DPPG. A more detailed explanation of the peptide-lipid interactions requires further investigations with applying structural techniques applicable to monolayers.

### 3.3. Time Evolution of Stationary Penetration Effects, ΔΠ versus *t* and ΔΔ*V* versus *t*

**Figure 4:**
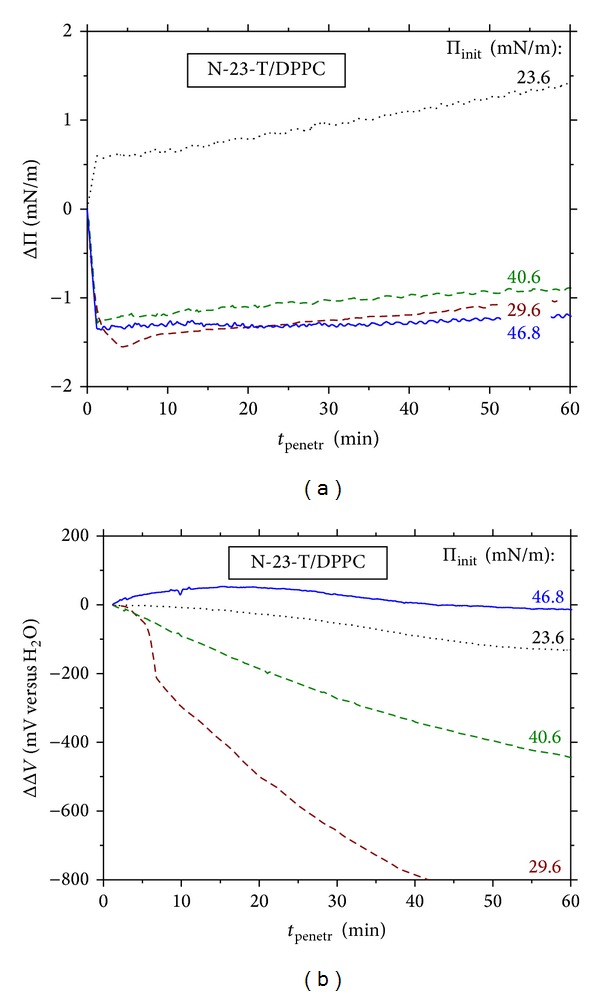
Time evolution of the effects on (a) surface pressure (ΔΠ) and (b) electric surface potential (ΔΔ*V*) caused by penetration of lactophoricin-I (N-23-T) to DPPC monolayer under stationary diffusion conditions; the courses registered at various initial densities of pure DPPC monolayer are characterized by the initial surface pressure values, Π_init_ (denoted therein). The peptide's concentration is 1 *μ*M.

**Figure 5:**
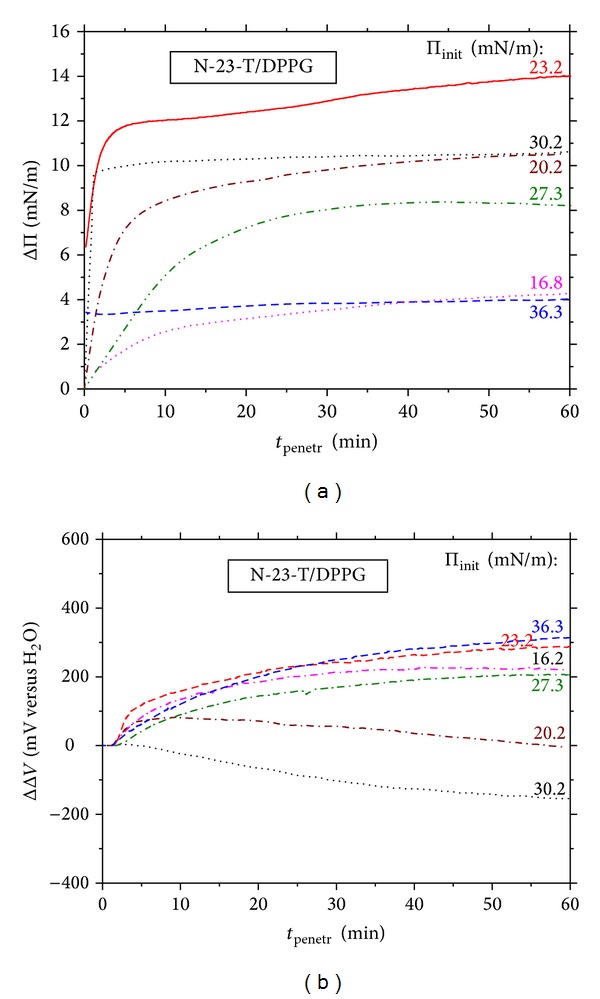
Time evolution of the effects on (a) surface pressure (ΔΠ) and (b) electric surface potential (ΔΔ*V*) caused by penetration of lactophoricin-I (N-23-T) to DPPG monolayer under stationary diffusion conditions; the courses registered at various initial densities of pure DPPG monolayer are characterized by the initial surface pressure values, Π_init_ (denoted therein). The peptide's concentration is 1 *μ*M.

Changes in the surface pressure (Π) and the electric surface potential (Δ*V*) during stationary penetration of N-23-T peptide into DPPC or DPPG monolayers are compared in Figures 4(a), 4(b), 5(a), and 5(b), respectively.

**Figure 6:**
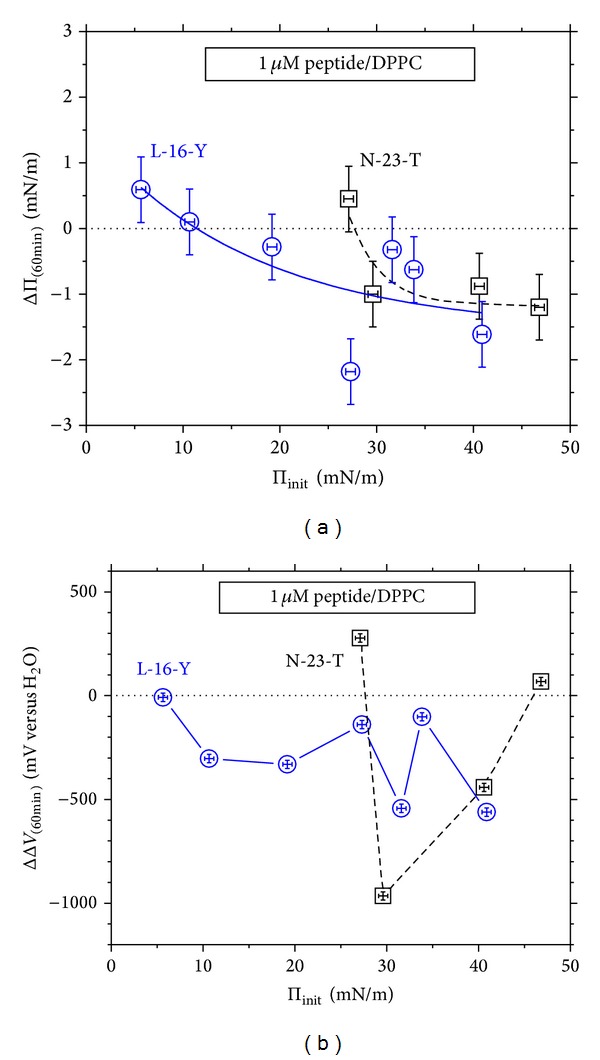
Effects of penetration of L-16-Y and N-23-T peptides to DPPC monolayer under stationary diffusion conditions on (a) surface pressure (Π) and (b) electric surface potential (Δ*V*); the ΔΠ and the ΔΔ*V* effects measured after 60 min. contacts are plotted as a function of density of the pure phospholipid monolayer characterized by the initial surface pressure, Π_init_. The error bar for Π_init_ and ΔΠ is ±0.5 mN m^−1^, and for Δ*V* is ±20 mV, at maximum. The peptides' concentration is 1 *μ*M.

One may see that stationary penetration of N-23-T into DPPC monolayers causes a negligible ΔΠ effect (Figures 4(a) and 6(a)), in contrast with that found from the compression isotherms (Figure 3). The small negative ΔΠ effects (of ca. −2.5 mN m^−1^, at maximum) were also measured during stationary penetration of the other peptides to DPPC monolayer. They indicate that the penetrants weaken lateral DPPC-DPPC interactions. The ΔΔ*V*-*t* courses measured for the DPPC/N-23-T system (Figure 4(b)) show a decrease of the Δ*V* up to ca. −1000 mV; the slightly less ΔΔ*V*-*t* decrease (by ca. −600 mV) was measured for the DPPC/L-16-Y system (results not shown). The negative ΔΔ*V* effect implies disorienting DPPC monolayer.

**Figure 7:**
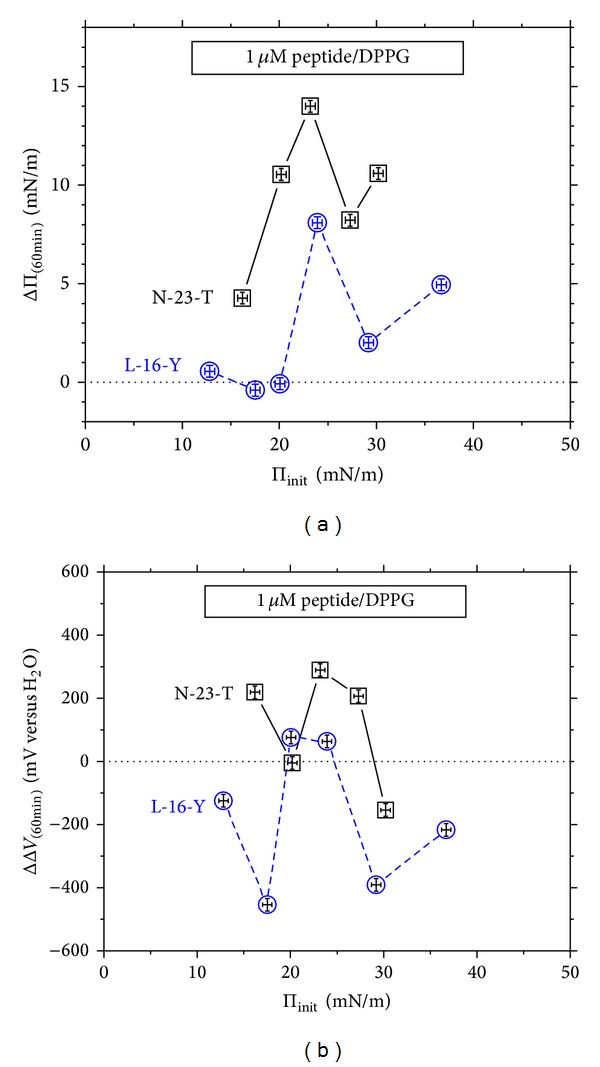
Effects of the stationary penetration of L-16-Y and N-23-T peptides into DPPG monolayer on (a) surface pressure (ΔΠ) and (b) electric surface potential (ΔΔ*V*); the effects measured after 60 min. contacts are plotted as a function of density of the pure phospholipid monolayer characterized by the initial surface pressure, Π_init_. The error bar for Π_init_ and ΔΠ is ±0.5 mN m^−1^, and for Δ*V* is ±20 mV, at maximum. The peptides' concentration is 1 *μ*M.

**Figure 8:**
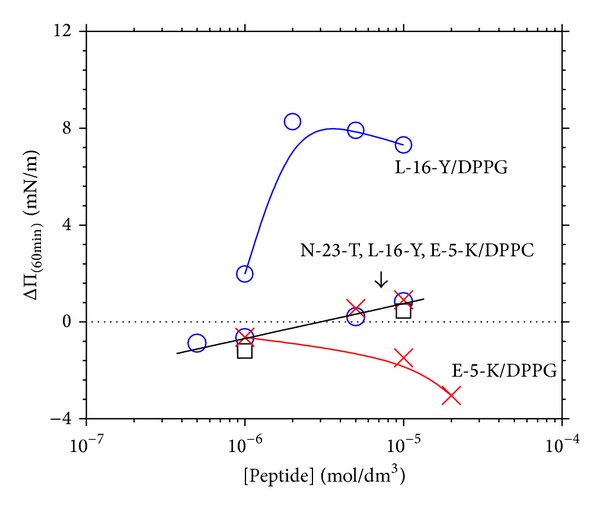
Effects of penetration of the different peptides to DPPC and DPPG monolayers on surface pressure (Π); The ΔΠ values measured under the stationary diffusion conditions after 60 min. contacts are plotted as a function of logarithm of peptide concentration. The initial surface pressure (Π_init_) of DPPC monolayers was in the range of 27.3 ± 0.3 mN m^−1^ and of DPPG—30.3 ± 0.3 mN m^−1^ (typical of density of biomembranes). The straight line corresponds to linear fit of the penetration effects of the three peptides to DPPC monolayer: ΔΠ_(60min⁡)_ = Π_(60 min⁡)_−  Π_init_ = *A* + *B* × log⁡ [Peptide] with *A* = 7.22 ± 0.75 mN m^−1^ and *B* = 1.30 ± 0.13 mN m^−1^ per decade of the peptide's concentration (*A* is of no physicochemical sense, since the results cannot be extrapolated to the extremely low concentrations). The error bars for ΔΠ (±0.5 mN m^−1^, at maximum) and for concentration (±5%), omitted herein for the sake of lucidity, correspond to diameter of the point mark.

The ΔΠ effects of the stationary penetration of the positively charged peptides to negatively charged DPPG monolayer (Figure 5(a)) are much greater as compared to DPPC; the maximum ΔΠ effect amounts to 14 mN m^−1^ for N-23-T (Figure 5(a)) and 8 mN m^−1^ for L-16-Y (Figure 7(a)). These ΔΠ effects fall within the range reported by other authors for penetration of various AMPs to monolayers of different phospholipids [48–50, 52, 53]. It should be emphasized here that affinities of N-23-T and L-16-Y for DPPG monolayer are comparable, despite the fact that N-23-T shows surface activity at the air/water interface, opposite to L-16-Y. (cf.  Section 3.1). It is reasoned by the fact, that, most of AMPs in a lipidic environment adopt an amphiphatic conformation which may be greatly different from that in the solution bulk [2, 11–19], as it was proven by circular dichroism (CD) and nuclear magnetic resonance (NMR) investigations [22, 23] (cf.  Table 1). The less negative effect of N-23-T on Δ*V* of DPPG monolayer, as compared to DPPC, is ascribed by us to the attractive, electrostatic peptide-DPPG interactions which drag the positive peptide into the monolayer on a greater depth.

### 3.4. Effects of Stationary Penetration as a Function of Phospholipid Density

The ΔΠ and ΔΔ*V* effects of stationary penetration of L-16-Y and N-23-T peptides to DPPC and DPPG monolayers, measured after the contact time of 60 min., are compared in Figures 6(a), 6(b), 7(a), and 7(b), as a function of the initial surface pressure of the monolayer, Π_init_.

It should be mentioned here that the hazardous step of the stationary penetration measurements (as mentioned in Section 2) is equalization of the penetrant's concentration immediately after injection, which may cause a disturbance of the phospholipid monolayer. On the other hand, manual operations in vicinity of the VP made vibrate may cause an uncontrolled shift in the apparatus zero level. (The vibrating plate technique is known of numerous interferences discussed by us in detail in [83]). Having the above in mind, the initial Δ*V* values against which the ΔΔ*V* effect was evaluated were taken just after finishing stirring the solution below the monolayer. The ΔΔ*V* effects evaluated on this way contain the Δ*V*-*t* evolution as a result of the preceding penetration, not including a stepwise change observed in some experiments during injection of peptide and equalization of its concentration. This mode of calculating the ΔΔ*V* effect assures the less as possible experimental error resulting only from accuracy of the Δ*V* measurement itself, ±10 mV, at maximum; this error shown by vertical error bars in Figures 6(b) and 7(b) falls within the used marks. The maximum error in determining Π_init_ values (taking into account the Π change during equilibration of the pure phospholipid monolayer and equalization of the peptide's concentration) was ±0.3 mN m^−1^. Since the error in determining Π_init_ values affects the ΔΠ = Π − Π_init_ dependence, the lateral and vertical error bars shown in Figures 6(b) and 7(b) are identical. As one can see in Figures 6(b) and 7(b), the experimental errors fall within the used marks with the exception of Figure 6(a).

The results presented in Figures 6(a) and 7(a) indicate nonlinearity of the ΔΠ versus Π_init_ dependencies, called the penetration profiles [56]. One can see that the ΔΠ versus Π_init_ profiles (Figures 6(a) and 7(a)) and the corresponding ΔΔ*V* versus Π_init_ dependencies (Figures 6(b) and 7(b)) show local extrema which of position on the Π_init_ axis depends on the phospholipid type. The extrema suggest a dependence of the penetration effects on 2D phase composition of the monolayer and, possibly, on shape and dispersion degree of the 2D phases' domains. Even though a number of authors have presented linear ΔΠ versus Π_init_ profiles for penetration of numerous AMPs [48–52] or bioactive peptides [47, 54, 55] to phospholipid monolayers, some other results suggest that linearity of ΔΠ versus Π_init_ is not the rule for all peptides [52, 53, 56–58]. For instance, Weroński and coworkers [58] obtained nonlinear ΔΠ versus Π_init_ dependencies for penetration of synthetic decapeptides (being a fragment of the nonstructural hepatitis G NS3 protein) to DPPC and DPPG monolayers. The profiles are of similar shape to those presented by us and show similar differentiation between DPPC and DPPG monolayers. Another example of ΔΠ versus Π_init_ profiles transferring throughout maximum is given in [56] for penetration of annexin-V to phospholipid monolayers formed with phosphocholine (POPC), phosphoglycerol (POPG), and phosphatidylserine (POPS), or their mixtures. On the other hand, a departure of some measuring points from the fitted linear dependence, ΔΠ versus Π_init_, [57] suggests a more complex course than the linear.

It is worth mentioning here that linear ΔΠ versus Π_init_ profiles presented in the literature for AMPs and bioactive peptides are mostly measured in range of Π_init_ below 30 mN m^−1^ [44, 49, 58] or much narrower [47, 54, 57]. In general, the linear ΔΠ versus Π_init_ profiles are used for determination of the so-called maximum insertion pressure (MIP) [47] (named also as the exclusion surface pressure) which is the extrapolated Π_init_ value at which no effect on the surface pressure occurs (ΔΠ = 0). This interpretation assumes that above the MIP no insertion of the peptide into the monolayer occurs. Since MIP values were usually found close to Π_init_ of 30 mN m^−1^ or less (while biomembranes are expected to show Π in the range of 30–35 mN m^−1^ [46, 47]), one could conclude from the results no ability of numerous AMPs to penetrate bacteria membranes in contrast to the documented antibacterial activities *in vitro*. Despite the fact that MIP values are useful for assessing potent interactions of bioactive peptides with biomembranes, one cannot directly refer them to penetration into phospholipid bilayers or bacteria membranes—the more advanced biomembrane model than a phospholipid monolayer. The other question concerning MIP was discussed by Barnes and coworkers [84, 85] who showed that some amphiphiles may exist in condensed Langmuir monolayers above MIP, on account for a high energy barrier of their ejection upon compression. The above mentioned ambiguities concerning MIP imply that a phospholipid monolayer upon compression (or decompression) does not reproduce all biomembrane properties determining penetration of AMPs. Nevertheless, the monolayer model of biomembranes is useful for investigating AMP-lipid interactions at initial stages of penetration under stationary diffusion conditions.

The profiles shown in Figures 6(a), 6(b), 7(a), and 7(b) let us follow conveniently sign of the ΔΠ and ΔΔ*V* effects in the particular systems. The stationary penetration to DPPC monolayers results in the ΔΠ and ΔΔ*V* effects negative in sign within almost the entire investigated Π_init_ range (Figures 6(a) and 6(b)). These ΔΠ versus Π_init_ profiles (Figure 6(a)) show a plateau in the Π_init_ range above 30 mN m^−1^, consistently for the two positively charged peptides, N-23-T and L-16-Y. This plateau implies reaching maximum of the low peptide interactions with DPPC monolayer. Simultaneously, the electric surface potential, Δ*V*, of DPPC monolayer is strongly lowered by the positively charged peptides, in particular at Π_init_ of ca. 30 mN m^−1^ (Figure 6(b)) which coincides with the greatest fluidity indicated by the *C*_*s*_^−1^ versus Π minimum (insert of Figure 1(a)).

On the other hand, stationary penetration of the positively charged peptides into DPPG monolayers produces ΔΠ effects positive in sign within almost entire investigated range of Π_init_ (Figure 7(a)), while the corresponding ΔΔ*V* effect shows reversal in sign along the Π_init_ axis (Figure 7(b)). The ΔΠ versus Π_init_ dependencies measured for the stationary penetration into DPPG monolayer (Figure 7(a)) show a narrow maximum at Π_init_ of ca. 24 mN m^−1^ which falls in the range of LC phase of pure DPPG monolayer (for discussion of 2D phase transitions in DPPG monolayers, see [62, 67–71]). The maximum ΔΠ effect indicates the maximum peptide-DPPG monolayer interactions in terms of lowering the surface free energy (the Π increase).

Notice that the total surface pressure of the discussed maximum, Π_init_ + ΔΠ = ca. 38 mN m^−1^, falls close to the range typical of real biomembranes and, on the other hand, within the region ascribed to the subsurface peptide-lipid interactions (cf. Figure 1(b)). The ΔΔ*V* maxima, occurring consistently at the same Π_init_ value as the ΔΠ maxima, suggest the best structural peptide-lipid adjustment (in terms of ordering molecular groups' dipoles which of greater inclinations to the interface contribute greater partial drops to the Δ*V*). Notice that the positive ΔΔ*V* effect prevails for N-23-T of the charge +3, while the negative ΔΔ*V* prevails for L-16-Y of the charge +4 (Figure 7(b)). It indicates that the positive ΔΔ*V* effect is not predominantly driven by the peptide's cationic charge. Even more, the greater cationic charge seems to cause a greater disorientation of DPPG monolayer as revealed by the greater lowering of the Δ*V* (Figure 7(b)). It may be explained by increasing depth of the penetration due to the stronger electrostatic interactions. The results of Figures 7(a) and 7(b) indicate that when DPPG density increases above that at which the ΔΠ versus Π_init_ maximum shows, both the specific interactions (indicated by ΔΠ) and structural fit ability of the peptide to DPPG monolayer (indicated by the ΔΔ*V*) diminish. These changes may be ascribed to decreasing depth of the peptide's penetration, followed by reorientation of phosphoglycerol groups.

### 3.5. Comparison between Stationary Penetration Effects and Those Evaluated from Compression Isotherms

It should be emphasized here that magnitude of the ΔΠ effects determined from the stationary penetration experiments (Figures 6(a) and 7(a)) differs notably from that evaluated from the compression isotherms (Figure 3). The difference is shown in particular for DPPC monolayer, for which the stationary penetration effect ΔΠ is negligible (of ca. −1 mN m^−1^, Figure 6(a)), while that evaluated from the compression isotherm is greater by order of magnitude (Figure 3). On the other hand, the stationary ΔΔ*V* effect reaches the maximum in the range of −500 and −1000 mV (Figure 6(b)), while that evaluated from the compression isotherms changes in the range between 30 and ca. −200 mV (Figure 3). The negligible ΔΠ effect obtained for the stationary penetration into DPPC monolayer coincides with a very low hemolytic activity (i.e., a negative effect on red blood cells, RBC) of N-23-T and L-16-Y. Comparing the data compiled in Table 1, one can see that N-23-T does not show hemolytic activity (which was checked at *c* ≤ 2 × 10^−4^ mol dm^−3^ [32]), while L-16-Y shows an insignificant one (i.e., 3.6% as compared to the effect of 1% Triton X-100 simulating 100% hemolysis [31]). It should be mentioned here that our investigations have been performed at much lower concentrations of the peptides than their minimum inhibitory concentrations (MIC) reported in the literature for various bacteria strains, that is, for N-23-T above 3 × 10^−4^ mol dm^−3^ [32], for L-16-Y in the range of 2.5 × 10^−5^–1 × 10^−4^ mol dm^−3^ [31, 32], and for E-5-K of ca. 5 × 10^−6^ mol dm^−3^ [26].

For the DPPG/N-23-T system, the ΔΠ effect evaluated from the compression isotherms is also notably greater than that of the stationary penetration, in particular, in the range of Π_init_ < 30 mN m^−1^. On the other hand, the corresponding ΔΔ*V* effect shows mostly positive in sign (i.e., below 250 mV, Figure 7(b)), while that evaluated from the compression isotherms is close to −20 mV, within almost entire range of Π_init_ > 3 mN m^−1^ (Figure 3). The stationary penetration results suggest that N-23-T exerts an orienting effect on DPPG monolayer, while the compression isotherms imply a slight disorienting effect in the range of Π_init_ > 3 mN m^−1^. It should be emphasized here that the two type experiments match reverse processes, as mentioned in Section 3.1. Results of the stationary penetration (own and the literature ones [47, 48]) indicate that stationary penetration in different lipid-peptide systems reaches the equilibrium after a time of hours. For this reason, one may presume that the reverse process (squeezing out of the monolayer) cannot reach the penetration equilibrium under the compression conditions usually used. This view is supported by the results [86] obtained for mixed monolayers of *β*-casein with phosphocholines (DPPC, DMPC, or DSPC) which show a strong dependence of the Π-*A* isotherm on equilibration time of a gaseous lipid film spread on subphase containing the peptide.

In conclusion, comparison between the penetration effects obtained by us from the two type experiments indicates that the effects measured upon monolayer compression correspond to a state of supersaturation of the monolayer with the penetrant and, simultaneously, to under-equilibrium orientation of the mixed monolayer. It should be stressed here that the ΔΠ effects calculated from the compression isotherms do not show the propensity of N-23-T for discriminating between negative and neutral lipid monolayers, while the stationary penetration experiments do. It is worth of mentioning here the insertion experiments (the Δ*A*/*A* versus *t* measurements) reported in [77] for penetration of the LL37 peptide into DPPC and DPPG monolayers which amply demonstrate the lipid head group discrimination by the LL37 while the Π-*A* isotherms do not reveal it. Indeed, the stationary penetration results are in accord with series of the literature reports on other AMPs [2, 5–21] which indicate their relatively strong interactions with phosphoglycerol groups in contrast to net neutral phosphocholine groups.

### 3.6. Stationary Penetration Effects as a Function of Peptide Concentration

The ΔΠ effects of penetration of the three peptides, N-23-T, L-16-Y, and E-5-K, to DPPC and DPPG monolayers are compared in Figure 8, as a function of logarithm of the penetrant's concentration, at Π_init_ close to 30 mN m^−1^.

One may notice the common linear ΔΠ versus log⁡ *c* dependency for penetration of the three peptides to DPPC monolayer which indicates the nonspecific interactions which probably occur through hydration shells. Such systems are describable with the Langmuir-Szyszkowski equation as discussed in relation to penetration of soluble amphiphiles to insoluble monolayers [63, 64]. Interestingly, the peptide-DPPC interactions are changing from slightly repulsive to slightly attractive ones with increasing the peptide's concentration above 3 × 10^−6^ mol dm^−1^. Since they do not correlate with structure of the peptides, nor with their charge, they may occur through the hydration shells of phosphocholine groups and of peptide in the subsurface region.

On the other hand, the ΔΠ versus log *c* dependencies measured for stationary penetration of L-16-Y and E-5-K peptides into DPPG monolayer show distinctly different nonlinear shape, indicating the specific peptide-lipid interactions (Figure 8). It is worth emphasizing here that penetration of E-5-K into DPPG monolayers results in a slight negative ΔΠ effect, within the entire investigated concentration range (Figure 8). This indicates the repulsive E-5-K/DPPG monolayer interactions which suggest a negative charge of E-5-K under the experimental conditions. A negative charge of E-5-K is also suggested by negative values of the Δ*V* at the air/solution interface (cf.  Section 3.1). According to theoretical prediction of IEP of E-5-K (cf.  Table 1), this peptide should adopt a slight positive charge at pH ca. 5.6. On the other hand, the net charge versus pH dependence (calculated using the “Peptide Property Calculator” of CS Bio Co.) indicates that E-5-K is neutral in the pH range of 5.5–8. The above inconsistencies may be ascribed to several reasons. First, there are some limitations in calculation of peptide's IEP owing to uncertainty as to p*K* values of some aminoacids which are regarded as independent for the calculation. For the above, IEP is usually calculated with the accuracy of ±0.5. Secondly, the actual pH at air/aqueous interface may essentially differ from that in the solution bulk. Taking the above in mind, the net charge of E-5-K under the conditions used may actually be slightly negative. On the other hand, the ΔΔ*V* effect produced by penetration of E-5-K into DPPC or DPPG monolayers (results not shown) was positive in sign, ranging from 50 to 300 mV. As was discussed for the positively charged peptides, sign of the ΔΔ*V* effect did not show a direct correlation with magnitude of the peptide charge; therefore, the ΔΔ*V* effect is primarily explained by us in terms of orienting the lipid. From this point of view, the relatively short E-5-K peptide, showing repulsive interactions with DPPG monolayer, may exert an orienting effect on it.

The processes investigated herein may be interpreted according to the first steps of the “carpet mechanism”, discussed in [2, 8, 9, 11–21] in relation to penetration of AMPs into phospholipid bilayers. At the first step, the penetrant binds to the matrix formed by the phospholipid head groups, not entering the acyl chains region. As it was shown by FTIR investigations, at a low peptide/lipid ratio, *α*-helical and *β*-sheet peptides orient parallel with their molecular axis towards the membrane's outer surface. When the ratio increases, the peptide begins to orientate in the membrane [1, 11, 13]. As was recently reported [87, 88], some AMPs possessing a hydrophobicity gradient along the *α*-helical long axis may penetrate the acyl chains region at a shallow angle, between 30° and 60°. The above mentioned stages may occur in the penetrated DPPG monolayer.

### 3.7. Long-Time Courses of Penetration, Π versus *t* and Δ*V* versus *t*

**Figure 9:**
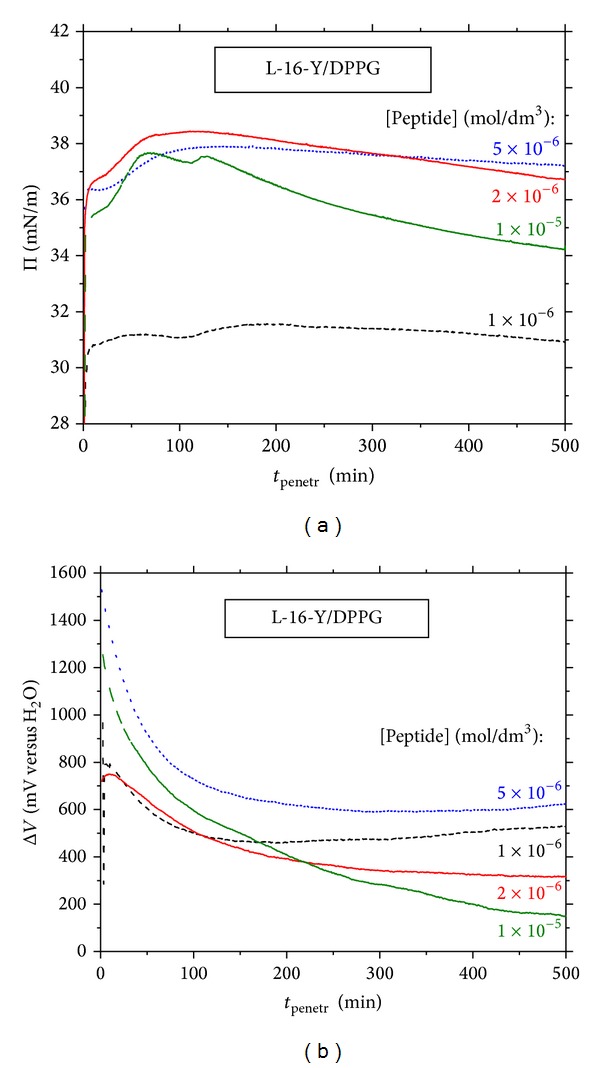
Long-time courses of (a) surface pressure, Π versus *t*, and (b) electric surface potential, Δ*V*versus *t*, owing to penetration of L-16-Y peptide to DPPG monolayers at various peptide concentrations (denoted therein). The initial surface pressure (Π_init_) of pure DPPG monolayer was 30 ± 0.3 mN m^−1^ (typical of density of biomembranes).

Figures 9(a) and 9(b) show representative Π-*t* and Δ*V*-*t* courses measured during ca. 10 hours of stationary penetration of L-16-Y to DPPG monolayer at different peptide's concentrations.

These results supply some essential information. First, the penetration equilibrium in DPPG monolayer is not established in real experiment time of 60 min, probably owing to a slow conformational equilibrium within the penetrated monolayer. Secondly, the long-time Π-*t* courses registered for the DPPG/L-16-Y system show non monotonic changes after 10–130 min of the contact (Figure 9(a)). They were revealed most distinctly at the highest peptide's concentration, 1 × 10^−5^ mol dm^−3^ (Figure 9(a)). These results suggest that the preceding peptide penetration into DPPG monolayer induces a 2D phase transition. In contrast, no non monotonic Π-*t* changes were observed during penetration of the peptides (L-16-Y and N-23-T) into DPPC monolayer (results not shown). This implies that only sufficiently strong peptide-lipid interactions (such as in case of DPPG monolayer) may induce 2D phase transitions during the proceeding penetration.

It should be stressed here that the Π-*t* courses illustrated in Figure 9(a) start at the Π_init_ close to 30 mN m^−1^; however, because of dependency of the ΔΠ effect on the peptide's concentration, the non monotonic changes fall at different Π values, between 31 and 38 mN m^−1^. This range coincides well with the upper limit of the wide *C*_*s*_^−1^ versus Π minimum found for DPPG monolayer on the subphase containing N-23-T (insert of Figure 1(b)), which may be ascribed to transformation of the peptide perturbed lipid domains into LC-DPPG domains, as a result of expulsing the penetrant to the subphase.

The corresponding long-time Δ*V*-*t* changes driven by the peptides penetration to DPPC or DPPG monolayers did not show discontinuities. The Δ*V*-*t* changes measured during the time period of ca. 10 hours exceed by several times those observed during the monolayer compression (cf. Figures 1(b) and 9(b)). The descending Δ*V*-*t* courses indicate progressive disorientation of phospholipid monolayer which may be the result of increasing the penetration depth. For the lowest peptide's concentrations, 1 × 10^−6^ and 2 × 10^−6^ mol dm^−3^, a local Δ*V*-*t* maximum was revealed (Figure 9(b)) indicating that, during the penetration process, the monolayer transfers throughout the relatively highest degree of ordering (ascribed by us to the best structural fitting of the peptide's and the lipid's orientations in the monolayer).

It should be mentioned here those the Π-*t* changes observed by us for the DPPG/L-16-Y system (Figure 9(a)) which show analogy to that described by Vollhardt and Fainerman [63] for penetration of *β*-lactoglobulin or *β*-casein to DPPC monolayer, and by Zhao and coworkers [89, 90]—for penetration of *β*-lactoglobulin to DPPC monolayer. The above mentioned authors combined observation of Π-*t* changes during the proceeding penetration (during 500 min) with periodically taken BAM and GIXD measurements. These investigations proved no specific interactions between DPPC molecules and *β*-lactoglobulin, or *β*-casein, which is in accord with our results for the milk-derived peptides. The above mentioned authors explained the noncontinuous Π-*t* change to LE/LC transition induced in DPPC monolayer by the penetrant (number and magnitude of domains observed with the BAM technique were notably dependent on initial density of DPPC monolayer). It is worth of mentioning here that similar long-time Π-*t* changes have been recently reported for stationary penetration of three peptides derived from human *β*-defensin to POPE-POPG (7 : 3) monolayers [91].

## 4. Conclusions 

Penetration of the positively charged antimicrobial, milk-derived peptides (N-23-T and L-16-Y) into DPPG monolayer, under stationary diffusion conditions, revealed nonlinear profiles of surface pressure, ΔΠ versus Π_init_, and electric surface potential, ΔΔ*V* versus Π_init_—the profiles transferring throughout a maximum at the Π_init_ of ca. 24 mN m^−1^, consistently. Quite distinctly different ΔΠ versus Π_init_ profiles were obtained for stationary penetration of the peptides into DPPC monolayer—they reach a minimum/plateau at the Π_init_ > 30 mN m^−1^. On the other hand, the ΔΠ effects evaluated by comparing the compression isotherms on pure water and N-23-T containing subphase are found much greater as compared to those measured under stationary diffusion conditions, in particular, for penetration into DPPC monolayer. In parallel, the ΔΔ*V* effects found from the compression isotherms are much less as compared to those measured upon the stationary penetration. What is more, the compression isotherms did not reveal such a great difference in affinity of lactophoricin-I for DPPG and DPPC monolayers, as it was found from the stationary penetration results. Comparison between our results obtained for lactophoricin-I from the two type experiments indicates that the ΔΠ and ΔΔ*V* effects measured upon compression of the penetrated monolayers (DPPC or DPPG) are much far from the equilibrium, both with respect to amount of the penetrant (which shows supersaturation) and to orientation of the monolayer (which shows an under-equilibrium orientation during compression). In fact, the only results obtained from the stationary penetration experiments are in accord with selective penetration of most antimicrobial peptides (AMPs) into negatively charged microbial membranes, as found *in vitro*. The principal conclusion of these experiments is that monolayer model of biomembrane when investigated under stationary diffusion conditions supplies results relevant to biological systems while the compression isotherms have to be interpreted with great caution.

The long-time Π-*t* courses (for ca. 10 hours) indicate that equilibrium of the penetration processes establishes after a time of hours, depending on the peptide/lipid ratio. This may be owing to a slow orientation/conformation change of the peptides associated with the lipid film. The Π-*t* courses measured during penetration of L-16-Y into DPPG monolayer show non continuous changes, notably dependent on the peptide's concentration. They indicate that the penetration process into DPPG monolayer induces 2D phase transitions driven by the sufficiently strong peptide-lipid interactions. No discontinuous changes were revealed during stationary penetration of the peptides into DPPC monolayer which is ascribed to the insignificant, repulsive peptide-DPPC interactions.

## Figures and Tables

**Table 1:** Data on structure and antimicrobial activities of the investigated peptides.

Name	Origin/molecular mass	Amino acid sequence	Total net charge at pH 7/pH 5.6^(a, e)^	Total hydrophobic ratio [%]^(a)^	No. of hydrophobic residues on molecule surface/all residues^(a)^	Secondary structure	IEP predicted	Antimicrobial and hemolytic activities
E-5-K	Residues 1–5 of *α*-lactalbumin/689 g/mol	H_2_N-EQLTK-COOH	0/ca. 0^(b)^	20	0/1	Random coil or quasi-pure *β*-sheet^(c)^ [26]	6.1 [26] 6.9 [35]	G. + bacteria; Not fungicidal [26]

L-16-Y	Residues 164–179 of *α*-casein/2011.4 Da	H_2_N-LKKISQRYQKFALPQY-COOH	+4/+4	31	0/5	Random coil [35] or *α*-helix^(c, e)^ [31, 35]	10.5 [35]	G. + and G. − bacteria; Slightly hemolytic [31]

N-23-T or Lactophoricin-I (LPcin-I)	Residues 113–135 of bovine component-3 of proteose peptone (PP3)/2683 Da	H_2_N-NTVKETIKYLKSLFSHAFEVVKT-COOH	+2.1/+3	39	9/9	*α*-helix^(c)^ [32], random in solutions and 50% helical content in LPC^(d)^ and SDS^(d)^ micelles [33], monomer in H_2_O [32]	9.4 [33] 9.95^(e)^	Mostly against G. + bacteria, not hemolytic [32]

^(a)^Values predicted using the “APD2: Antimicrobial Peptide Predictor” software, available at http://aps.unmc.edu/AP/main.php [3].

^(b)^According to the theoretical prediction of IEP of E-5-K (consistent with [26, 35]), this peptide should adopt a slightly positive charge at the used pH ca. 5.6; however, the penetration results (Figure 8) indicate a slight negative charge of E-5-K, under the experimental conditions.

^(c)^Derived according to the secondary structure algorithm [31].

^(d)^LPC denotes L-*α*-lysophosphatidylcholine, and SDS denotes sodium dodecyl sulphate.

^(e)^The value is predicted using the “Peptide Property Calculator” of CS Bio Co. software available at http://pepcalc.com/ppc.php (used also in [35]).
